# Investigating the Interplay between Olfaction, Social Behaviour, and Individual Differences: Editorial

**DOI:** 10.3390/brainsci14020142

**Published:** 2024-01-30

**Authors:** Laiquan Zou, Mehmet K. Mahmut

**Affiliations:** 1 Department of Psychology, School of Public Health, Southern Medical University, Guangzhou 510515, China; zoulq@smu.edu.cn; 2 School of Psychological Sciences, Macquarie University, Sydney, NSW 2109, Australia

## 1. Introduction

Long-COVID mercilessly brought awareness to what olfaction researchers have known all along; losing your sense of smell changes your experiences with the world around you and can compromise your health and wellbeing [1,2,3]. Therefore, the aim of our Special Issue is to highlight the intricate and unexpected ways our sense of smell may be linked to our thoughts, emotions, and behaviours. The sense of smell predicts numerous facets of human behaviour in various ways; for example, higher odour acuity is associated with a larger social network [4], single men’s body odour smells stronger and sexier than partnered men’s body odour, and olfactory loss can be predictive of Parkinson’s disease [5]. We invited researchers to submit studies that investigate the interplay between physiology and psychology and demonstrate how the interaction between our sense of smell and individual differences may influence, motivate, and predict human behaviour. We are delighted to have published 10 articles plus one comment in this Special Issue and will take this opportunity to summarise the findings of our contributors’ work, listed below, and provide a summary of the articles included based on the five main themes that emerged: (1) the social and psychological correlates of olfaction, (2) olfaction and mental health, (3) olfactory physiology and function, (4) the impact of COVID-19 on memory, and the (5) neuroanatomical hypothesis of olfactory function.

## 2. An Overview of the Published Articles

### 2.1. Social and Psychological Correlates of Olfaction

De Groot and colleagues (contribution 1) cleverly combined consumer psychology, the chemical senses, and sustainability in this novel study that investigated whether a fresh laundry odour imbued on clothes in a second-hand store would increase sales compared to clothes with no odour or a citrus odour. The results (based on almost 7000 transactions) indicated that significantly more money was spent when clothes had a fresh laundry odour compared to the other two conditions. These findings highlight the impact that our sense of smell has on our behaviour and how it may be harnessed to enhance environmental sustainability.

We were privileged to have the contribution of Professor Theresa White, and colleagues (contribution 2), a researcher who inspired MKM’s first thesis on olfactory memory. In their contribution, Professor White and colleagues presented a study investigating whether language influences the perception of household odours as gendered by comparing native French (a grammatically gendered language) to native English (a non-grammatically gendered language) speakers. The results revealed that both native-French and native-English speakers produced adjectives describing odours consistent with their semantic gender (e.g., onion is anthropomorphised as a masculine smell so may be associated with stereotypically masculine adjectives like strong and dominant). Moreover, a bias for describing household odours as feminine was found with both speaker groups, although this may be due to nature of the odours used, rather than exclusive or explicit odour–gender associations. This research speaks to the broader impact culture can have on our vocabulary for and semantic association with odours.

Keaveny and Mahmut (contribution 9) explored, for the first time, whether anecdotal reports that some women find their partner’s body odour disgusting during the breakdown of their relationship can be confirmed empirically. Partnered (n = 48) and single, previously partnered women (n = 32) completed surveys regarding their relationship satisfaction and commitment, exposure to their current/former partner’s various body odours (e.g., sport sweat, breath, and feet) plus the three Sniffin’ Sticks tests. The results indicated that participants who experienced a relationship breakup reported higher hedonic (e.g., sexiness) ratings of their previous partner’s body odours. Moreover, currently partnered participants with lower relationship commitment were more likely to report a desire to breakup with their partner after smelling their various body odours. The researchers theorise that repeated exposure to one’s partner’s body odours may reflect or solidify relationship commitment but that further research is required to determine whether psychological disgust for one’s partner precedes psychophysiological disgust for their body odours.

The final results come from Shell and colleagues’ (contribution 10) who conducted a self-report-based study exploring the link between attachment style and olfactory ability. Four-hundred and one participants (mostly female) completed a series of surveys measuring their attachment tendencies, experiences, and satisfaction within romantic relationships, awareness of and attention to odours, and a self-assessment of their olfactory ability. The results revealed that attachment insecurity (both anxious and avoidant) was associated with poorer olfactory ability for females. The researchers theorise that smelling a partner’s body odour may activate attachment systems and facilitate one mode of feeling connected to their partner, something that those with a poorer sense of smell may miss out on.

### 2.2. Olfaction and Mental Health

Feng and Lei (contribution 4) presented findings on the interplay between visual and olfactory perception. Specifically, they presented participants with morphed faces varying in attractiveness paired with either a “non-social” pleasant odour (i.e., orange), “non-social” unpleasant odour (i.e., valeric acid, often perceived as a stale cheese/vomit-like odour), and a neutral odour (i.e., water). The researchers found that faces paired with the pleasant or neutral odour were rated more attractive than those presented with the unpleasant odour and that, overall, male faces were rated as more attractive than female faces. The authors conclude that their findings provide preliminary evidence that cross-modal integration of visual and olfactory stimuli can influence perceptions of important human features such as faces.

Lübke and colleagues (contribution 5) presented the results of a study investigating whether greater olfactory ability is associated with an increased ability to infer another person’s emotions. Among other measures, participants completed the Düsseldorf Odour Discrimination Test and a task requiring them to indicate, solely by looking at images of eye regions, what the person was feeling from a list of four options. Their results demonstrated that for women only, higher odour discrimination ability was significantly correlated with higher emotion recognition. These findings are consistent with previous findings that better olfactory ability is associated with higher levels of empathy and that females have higher levels of empathy [6,7].

Chen and colleagues (contribution 6) explored for the first time in children and adolescents (9–16 years) whether olfactory ability, schizotypal traits, and hedonic ratings of odours were connected. Specifically, 355 non-clinical participants completed an odour identification test (Universal Sniff Test), a self-report measure of how much pleasure is derived from odours and tastes, and a self-report measure of schizotypal traits. The results showed that higher degrees of schizotypal traits were associated with poorer odour identification ability and a lower capacity to experience pleasure from odours. Moreover, odour identification ability mediated the relationship between odour hedonic capacity and schizotypal traits. The authors conclude with a novel approach to improving odour hedonic capacity, that is, by engaging in olfactory training with the aim of improving odour identification ability. 

Liu and colleagues (contribution 7) presented their research findings on dementia risk in late-life depression based on odour identification ability. One-hundred and seventy-nine participants with late-life depression and 189 controls completed a series of neuropsychological tests plus the Sniffin’ Sticks odour identification test. They found that participants with late-onset depression had significantly lower odour identification scores than those with early-onset depression and controls. Moreover, the difference in odour identification ability between early- and late-onset depression was driven by the latter’s poorer memory and language function. The researchers highlight that many neural regions are involved in odour identification ability and further studies are required with age-matched groups before odour identification ability can be used as a predictor of dementia in late-life depression.

### 2.3. Olfactory Physiology and Function

Yan and colleagues (contribution 8) presented MRI findings on individual differences in olfactory bulb shape and olfactory function. One-hundred and ninety-two participants with the most common olfactory dysfunctions and 77 age- and gender-matched controls completed the extended Sniffin’ Sticks and had an MRI scan of their olfactory bulb. The olfactory bulb was categorized in terms of its volume (mm^3^) and convexity and shape (i.e., convex: olive, circle, or plano-convex; non-convex: banana, irregular, plane or scattered). The results revealed that non-convex olfactory bulb shapes were significantly more likely to occur in olfactory dysfunction patients than in controls. Moreover, those with olive-shaped olfactory bulbs had the highest scores on the Sniffin’ Sticks and those with plane-shaped olfactory bulbs had the lowest scores. Finally, their results also indicated that olfactory bulb shape changes with age. 

### 2.4. Impact of COVID-19 on Memory

Fiorentino and colleagues (contribution 3) presented the results of a study with numerous neuropsychological tests completed by participants with persistent COVID-19 symptoms. They found that a quarter of their sample reported psychological distress, 20% reported fatigue, and 20% qualified for the classification of functional anosmia (Sniffin’ Sticks total score ≤16). Neuropsychological testing revealed that 20% of the participants evidenced impaired (written verbal) semantic memory and that poorer performance on the TODA threshold test was associated with poorer semantic memory. The authors indicate that their findings support neuropsychological deficits associated with the temporal lobe, in particular, hippocampal structures within the left temporal lobe. The authors’ work reflects that the fact that viruses impacting neural structures and pathways involved in olfaction will not solely manifest as olfactory deficits due to the overlapping nature of their roles.

### 2.5. Neuroanatomical Hypothesis of Olfactory Function

In a comment on Fiorentino and colleagues’ findings (contribution 3), De Luca and colleagues (contribution 11) postulated the process by which the SARS-CoV-2 virus may cause memory deficits, specifically, in which neuro-inflammation may spread from the entorhinal cortex to the hippocampus.

## 3. Conclusions and Directions for Future Research

The articles published in this Special Issue have provided exciting new insights into the relationship between olfaction, physiology, and psychology. To continue unveiling the connection olfaction shares with various aspects of human behaviour, future research should endeavour achieve the following. First, future studies should widen the generalisability of the findings by increasing the diversity of the samples (e.g., age, sex, gender, clinical status, and geographic location). Sample diversity is particularly important given that olfactory function can differ based on age, sex, environment, and the findings of such olfaction. Second, given that many of the findings reported here are novel, a replication of the results is required to ensure their validity. Third, while some non-clinical olfaction studies conclude that their findings may have therapeutic implications, very few studies have tested such hypotheses. Finally, few empirical studies have attempted to determine the causality of any relationships found between olfactory function and human behaviour, leaving a large untapped field of research. Understanding the causal mechanisms of any relationships olfaction bears with human psychology and/or physiology is particularly important if researchers are to realise the potential therapeutic implications of their findings.
